# Zinc-Induced PKCδ-dependent Phosphorylation of MTF-1 Promotes Pulmonary Vascular Remodeling in Hypoxic Pulmonary Hypertension

**DOI:** 10.7150/ijbs.124664

**Published:** 2026-01-01

**Authors:** Ai Chen, Yan Yan, Rong Cao, Kexin Cai, Guili Lian, Wenqin Cai, Jianfu Zhou, Liangdi Xie

**Affiliations:** 1 Department of Geriatrics, Fujian Hypertension Research Institute, the First Affiliated Hospital, Fujian Medical University, Fuzhou 350005, China.; 2 International Medical Department, National Regional Medical Center, Binhai Campus of the First Affiliated Hospital, Fujian Medical University, Fuzhou 350212, China.; 3 Department of Cardiology, the First Affiliated Hospital, Fujian Medical University, Fuzhou 350005, China.; 4 Fujian Key Laboratory of Precision Medicine for Cancer, the First Affiliated Hospital, Fujian Medical University, Fuzhou 350005, China.

**Keywords:** pulmonary hypertension, zinc, metal-responsive transcription factor 1, phosphorylation, protein kinase Cδ, APTO-253

## Abstract

Pulmonary hypertension (PH) is driven by pulmonary vascular remodeling, in which the zinc-sensing transcription factor metal-responsive transcription factor 1 (MTF-1) may play a pivotal regulatory role. Rodent models of hypoxia-induced PH and cultured pulmonary arterial smooth muscle cells (PASMCs) were used to investigate zinc-mediated MTF-1 activation. Phos-tag SDS-PAGE, site-directed mutagenesis, Cleavage Under Targets and Tagmentation (CUT&Tag), and pharmacological inhibitors were employed to dissect the PKCδ/MTF-1/placental growth factor (PlGF) pathway. CUT&Tag profiling revealed prominent MTF-1 enrichment at promoter regions under hypoxia, with significant occupancy at the *Plgf* locus and enrichment of angiogenesis-related genes. Hypoxia increased intracellular zinc levels, activated PKCδ, and triggered phosphorylation of MTF-1 at Ser^304^. This modification was essential for MTF-1 nuclear translocation and PlGF transcription. Mutation of Ser^304^ or MTF-1 knockdown suppressed PASMCs proliferation and migration under hypoxia and zinc exposure. Gö 6983 abrogated MTF-1 phosphorylation and downstream responses, and selective knockdown of PKCδ reproduced these effects, confirming PKCδ as the predominant isoform mediating MTF-1 activation. *In vivo*, MTF-1 and PlGF were upregulated in pulmonary vessels of Su/Hx-PH rats, while APTO-253 treatment attenuated pulmonary vascular remodeling and improved cardiopulmonary hemodynamics in hypoxic mice. This study identified PKCδ-dependent phosphorylation of MTF-1 at Ser^304^ as a critical mechanism linking zinc accumulation to PlGF-driven PASMCs proliferation. Targeting the zinc/PKCδ/MTF-1/PlGF axis represented a novel therapeutic strategy for hypoxic PH.

## 1. Introduction

Pulmonary hypertension (PH) is a severe and progressive cardiopulmonary disorder defined by sustained elevation of pulmonary arterial pressure, leading to maladaptive vascular remodeling and eventual right heart failure^1^. Among the pathological features of PH, excessive proliferation and resistance to apoptosis of pulmonary arterial smooth muscle cells (PASMCs) are central to the occlusive remodeling of small pulmonary arteries and arterioles^2^. While the molecular drivers of PASMCs hyperplasia remain incompletely understood, growing evidence points to aberrant metal ion signaling, particularly zinc, as a contributing factor in vascular remodeling^3^, ^4^.

Zinc is an essential trace element involved in numerous cellular processes, including proliferation, oxidative stress responses, and transcriptional regulation^5^. In previous work, we demonstrated that disruption of zinc balance exacerbates monocrotaline-induced PH in rodent models^6^, in part through activation of metal-responsive transcription factor 1 (MTF-1) and its downstream target, placental growth factor (PlGF), a pro-angiogenic and mitogenic cytokine^7^, ^8^. MTF-1-mediated PlGF induction promoted PASMCs proliferation and contributed to pulmonary vascular remodeling^9^. However, whether this zinc/MTF-1/PlGF signaling axis is similarly activated in hypoxia—a major etiological factor in PH—remains unknown.

MTF-1 is a highly conserved transcriptional regulator^10^ that remains cytoplasmic under basal conditions but translocates to the nucleus in response to cellular stress, including hypoxia^11^, ^12^. While bioinformatic analyses have predicted phosphorylation sites within MTF-1^13^, ^14^, the role of phosphorylation in regulating its activity in the context of hypoxic PH has not been elucidated.

In this study, we investigated the functional relevance of the zinc/PKCδ/MTF-1/PlGF axis in hypoxia-induced PH. We examined intracellular zinc dynamics and MTF-1 activation in rodent models of hypoxic PH and used Phosphate-binding tag Sodium Dodecyl Sulfate Polyacrylamide Gel Electrophoresis (Phos-tag SDS-PAGE) to identify critical phosphorylation sites within MTF-1. Furthermore, we assessed the therapeutic potential of APTO-253, a selective MTF-1 inhibitor, in modulating PASMCs proliferation and vascular remodeling under hypoxic conditions. Our findings identify a previously unrecognized mechanism linking zinc signaling to hypoxia-driven vascular pathology and suggest MTF-1 inhibition as a promising therapeutic strategy for PH.

## 2. Materials and Methods

### 2.1 Animals and PH models

All animal procedures were approved by the Institutional Animal Care and Use Committee of the First Affiliated Hospital of Fujian Medical University and conducted in accordance with institutional guidelines. To establish the Sugen 5416/hypoxia-induced pulmonary hypertension (Su/Hx-PH) model, male Sprague-Dawley rats (6-8 weeks old, 180-200 g) received a single subcutaneous injection of Sugen 5416 (20 mg/kg; Sigma-Aldrich, United States), which is the widely accepted standard dose for this model as reported in prior studies^15^, ^16^, followed by continuous exposure to hypoxia (10% O_2_) for 3 weeks in a hypoxic chamber. Rats were then returned to normoxic conditions (21% O_2_) for an additional 2 weeks. For the hypoxia-induced PH (Hx-PH) mouse model, adult male C57BL/6 mice (6-8 weeks old, 18-30 g) were housed in normobaric hypoxia chambers (10% O_2_; Yuyan Instruments, China) for 5 consecutive weeks. To assess the therapeutic efficacy of MTF-1 inhibition, a subset of hypoxic mice received intraperitoneal injections of APTO-253 (15 mg/kg), a mid-range dose adapted from previous rodent studies demonstrating efficacy and safety in the 10-20 mg/kg range^17^-^19^. The drug was administered twice weekly (on days 1 and 2) throughout the 5-week hypoxia exposure period (Hx-PH + APTO). Normoxic controls (Ctrl) were housed under ambient air (21% O_2_) with otherwise identical conditions.

At the study endpoint (week 5), hemodynamic parameters were evaluated. In rats, pulmonary arterial pressure (PAP) and right ventricular pressure (RVP) were measured via right heart catheterization through the external jugular vein using a micro-catheter advanced into the pulmonary artery. In mice, RVP was measured via closed-chest transthoracic intercostal puncture. Hemodynamic signals were recorded and analyzed using a PowerLabacquisition system and LabChart software (AD Instruments, Australia). Following measurements, hearts were harvested and dissected to determine the right ventricular hypertrophy index (RVHI), calculated as the ratio of right ventricular weight to the sum of left ventricular and septal weight [RV/(LV+S)].

Lung tissues were collected for histopathological analysis. Sections were subjected to hematoxylin and eosin (H&E) staining, immunohistochemistry (IHC), and immunofluorescence (IF) to assess pulmonary vascular remodeling. Morphometric measurements were performed on small pulmonary arteries, and vessel wall parameters were quantified as follows: wall thickness percentage (WT%) = (wall thickness / external diameter) × 100; wall area percentage (WA%) = (wall area / total vessel area) × 100.

This study was performed in line with the principles of the Declaration of Helsinki. Approval was granted by the Ethics Committee of Fujian Medical University (Approval No. 2017-070, Fuzhou, China).

### 2.2 Primary PASMCs isolation and culture

Primary PASMCs were isolated from male Sprague-Dawley rats (6-8 weeks old) as previously described, with minor modifications. Briefly, intrapulmonary arteries were dissected under sterile conditions and enzymatically digested using collagenase and elastase to obtain a single-cell suspension. Cells were cultured in Dulbecco's Modified Eagle Medium (DMEM; Hyclone, United States) supplemented with 10% fetal bovine serum (FBS; Gibco, Australia) and maintained at 37 ℃ in a humidified incubator with 5% CO_2_. All experiments were conducted using cells between passages 2 and 4. To simulate hypoxic stress, PASMCs were treated with cobalt chloride (CoCl_2_, 100 μM) for 24 h. For stimulation and pharmacological assays, cells were exposed to zinc sulfate (ZnSO_4_, 100 μM) or the selective MTF-1 inhibitor APTO-253 (10 μM) under identical conditions. Vehicle controls were included as appropriate.

### 2.3 Western blot and subcellular fractionation

Cytoplasmic and nuclear protein fractions were isolated using a Nuclear and Cytoplasmic Protein Extraction Kit (Beyotime, China) according to the manufacturer's instructions. Briefly, cells were collected on ice, lysed, and centrifuged at 12,000 rpm for 10 min. Equal amounts of protein were separated by 10% SDS-PAGE and transferred to PVDF membranes. Membranes were blocked in 5% non-fat milk for 1 hour at room temperature and incubated overnight at 4 ℃ with primary antibodies. β-Actin and Lamin B served as loading controls for cytoplasmic and nuclear proteins, respectively. Following incubation with HRP-conjugated secondary antibodies (1:8000, RS020103/YM0337, Immunoway, China), immunoreactive bands were visualized using enhanced chemiluminescence (ECL; Thermo Fisher, United States) and imaged with a digital gel documentation system. Band intensities were quantified by densitometric analysis using ImageJ software.

β-Actin and Lamin B were used as loading controls for cytoplasmic and nuclear fractions, respectively. The following primary antibodies were used: MTF-1 (1:500, custom-made, HuaBio Biotech, China), PCNA (1:500, ab18197, abcam, United States), PlGF (1:500, sc-518003, Santa Cruz, United States), β-actin (1:2000, sc-47778, Santa Cruz, United States), Lamin B (1:1000, sc-6217, Santa Cruz, United States), T-PKC (1:500, sc-937, Santa Cruz, United States), pPKC (1:500, HA722458, HuaBio Biotech, China), pPKCδ (1:500, #9374, Cell Signaling Technology, United States), KLF4 (1:500, R1308-1, HuaBio Biotech, China), Cyclin D1 (1:500, ET1601-31, HuaBio Biotech, China).

### 2.4 Immunoprecipitation assay and phosphate-binding tag (Phos-tag) SDS-PAGE

For co-immunoprecipitation (co-IP) assays, HEK293T cells or primary PASMCs were transfected with Flag-tagged MTF-1 expression plasmids using Lipofectamine 3000 (Thermo Fisher, United States) according to the manufacturer's protocol. Forty-eight h post-transfection, cells were lysed in IP lysis buffer (Beyotime, China) supplemented with protease and phosphatase inhibitors. Lysates were incubated with anti-Flag M2 affinity agarose beads (Sigma-Aldrich) overnight at 4 ℃ with gentle rotation. After extensive washing with lysis buffer, bound proteins were eluted with SDS loading buffer, denatured at 95 ℃ for 5 min, and subjected to Western blot analysis.

Phos-tag SDS-PAGE was performed to assess MTF-1 phosphorylation. Cell lysates were resolved on 8% SDS-PAGE gels containing 50 µM Phos-tag acrylamide and 100 µM MnCl_2_ (Wako Pure Chemical Industries, Japan). Following electrophoresis, gels were equilibrated in transfer buffer supplemented with 10 mM EDTA for 10 min to chelate Mn^2+^ ions, and proteins were transferred to PVDF membranes. Membranes were probed with anti-MTF-1 antibody (1:500, HuaBio Biotech, China) and developed using enhanced chemiluminescence. To verify phosphorylation specificity, parallel lysates were treated with calf intestinal alkaline phosphatase (CIAP, 1 U/µg protein, 30 min at 37 °C) before electrophoresis.

### 2.5 Reverse transcription-quantitative polymerase chain reaction (RT-qPCR)

Total RNA was extracted using TRIzol (Vazyme, China) according to the manufacturer's protocol. Complementary DNA was synthesized with M-MLV reverse transcriptase (Vazyme, China) from 1 µg of total RNA. Quantitative PCR was performed on a Roche Real-Time PCR System using SYBR Green chemistry. Gene expression levels were normalized to *Gapdh*, and relative mRNA abundance was calculated using the 2^-ΔΔCt^ method. Primer sequences are provided in **Supplementary Table 1**.

### 2.6 Cell proliferation and migration assays

PASMCs proliferation was evaluated using Cell Counting Kit-8 (CCK-8), 5-ethynyl-2'-deoxyuridine (EdU) incorporation, and flow cytometry-based cell cycle assays.

For the CCK-8 assay, cells were seeded into 96-well plates at a density of 5 × 10^3^ cells per well in 100 μL medium. After treatment, 10 μL of CCK-8 reagent was added and incubated for 2 h. Absorbance at 450 nm was measured using a microplate reader (BioTek, United States). Background absorbance from wells containing medium plus CCK-8 reagent but no cells was subtracted from all readings. Linearity of the assay was verified in pilot experiments by seeding serial dilutions of PASMCs (1 × 10^3^ to 2 × 10^4^ cells/well) and confirming proportionality of OD values.

For the EdU assay, PASMCs were seeded into 24-well plates at 3 × 10^4^ cells per well, incubated with 10 μM EdU for 4 h, fixed (4% paraformaldehyde), permeabilized (0.5% Triton X-100), and counterstained with DAPI (1:1000, Cell Signaling Technology, United States). Images were acquired on a fluorescence microscope (Nikon, Japan) using a 20× objective; five random fields/well were captured with identical exposure. At least 500 cells/condition were counted and the EdU^+^/DAPI^+^ fraction calculated. Quantification was blinded and performed in ImageJ 1.54f (National Institutes of Health, Bethesda, MD, United States).

For flow cytometry cell cycle analysis, PASMCs were harvested, washed twice with cold PBS, and fixed in 70% ethanol at -20 ℃ overnight. Fixed cells were washed and incubated with RNase A (100 μg/mL) at 37 ℃ for 30 min to remove RNA, followed by staining with propidium iodide (PI, 50 μg/mL) in the dark. Data were acquired on a Beckman Coulter CytoFLEX S cytometer. For each sample, ≥10,000 events were collected. Doublets were excluded by gating on PI area versus PI width, and cell cycle distribution was analyzed using CytExpert software (Beckman Coulter, United States).

Cell migration was assessed using a scratch assay. PASMCs were seeded into 6-well plates at 2 × 10^5^ cells per well and grown to full confluence. A uniform linear scratch (~700-800 μm) was made using a sterile 200 μL pipette tip, and wells were washed twice with PBS to remove detached cells. To minimize confounding effects of proliferation, cells were pre-treated with mitomycin C (10 μg/mL, 2 h) and assays were performed in 1% FBS medium. Images were acquired immediately (0 h) and after 48 h using the same microscope settings, using a 10× or 20× objective. The scratch area was quantified in ImageJ, and migration was expressed as the percentage wound closure: *(scratch width at 0 h - scratch width at 48 h) / scratch width at 0 h × 100*. Five random fields/well were analyzed by blinded investigators.

### 2.7 Immunofluorescence (IF) staining

PASMCs were fixed with 4% paraformaldehyde for 15 min at room temperature and permeabilized with 0.2% Triton X-100 in PBS for 10 min. After blocking with 5% bovine serum albumin (BSA) for 1 h, cells were incubated overnight at 4 ℃ with anti-MTF-1 primary antibody (1:200, HuaBio Biotech, China). After washing, cells were incubated for 1 h at room temperature in the dark with Alexa Fluor 488-conjugated goat anti-rabbit IgG secondary antibody (1:500; Invitrogen, United States). Nuclei were counterstained with DAPI (1 μg/mL, 5 min). Images were acquired using a Zeiss LSM880 confocal microscope with a 63× oil immersion objective. Excitation/emission settings were 488/520 nm for Alexa Fluor 488 and 405/460 nm for DAPI. Z-stacks (0.5 μm intervals, 6-8 planes per cell) were collected and maximum intensity projections generated for quantification. For quantification, five randomly selected fields per sample and at least 100 cells per condition were analyzed. Nuclear-to-cytoplasmic fluorescence intensity ratios were calculated using ImageJ 1.54f (National Institutes of Health, Bethesda, MD, United States) with the “ROI Manager” tool. The workflow included manual outlining of nuclear and cytoplasmic regions, measurement of mean fluorescence intensity, and calculation of nuclear/cytoplasmic ratios. Analysis was performed by two independent blinded observers. Negative controls included omission of the primary antibody and isotype IgG control staining to confirm signal specificity.

### 2.8 Plasmids and siRNA transfection

Plasmids encoding wild-type MTF-1, PlGF, and MTF-1 phosphorylation site mutants (S5A, S151A, T253A, S304A, S304E) were synthesized by GenePharma (**Supplementary Table 2**). Site-directed mutagenesis was performed to substitute serine/threonine residues with alanine (A; to mimic dephosphorylation) or aspartic acid (E; to mimic constitutive phosphorylation). Corresponding siRNAs targeting *Mtf-1*, *Plgf* and *Prkcd* were also obtained from GenePharma, and sequences are provided in **Supplementary Table 3**.

HEK293T cells and primary PASMCs were seeded into 6-well plates and transfected with plasmids or siRNAs using Lipo8000 reagent (Beyotime, China) according to the manufacturer's instructions. For each well, 2.5 μg plasmid DNA or 100 nM siRNA was used. After 48 h, cells were harvested for subsequent analyses, including immunoblotting and dual-luciferase reporter assays.

### 2.9 Dual-luciferase reporter assay

The *Plgf* promoter sequence (-800 to +100 bp relative to the transcription start site), containing two canonical metal-responsive elements (MREs), was synthesized by GenePharma (Shanghai, China) and inserted upstream of the firefly luciferase gene in the pGL4.20 vector. Mutant reporter constructs were generated by site-directed mutagenesis, in which the core MRE motifs (TGCRCNC) were replaced with TTTTTT to abolish MTF-1 binding. This design allowed evaluation of MTF-1-dependent transcriptional activation of the *Plgf* promoter under different stimulation conditions.

HEK293T cells were co-transfected with pGL4.20-*Plgf*-luc (wild-type or mutant), a Renilla luciferase control plasmid (pRL-TK), and either pcDNA3.1-*Mtf-1* or empty vector using Lipo8000 reagent (Beyotime, China) according to the manufacturer's protocol. Twenty-four hours post-transfection, cells were exposed to the indicated treatments. After an additional 24 h, cells were lysed, and firefly and Renilla luciferase activities were measured using the Dual-Lumi II Luciferase Reporter Gene Assay Kit (Beyotime, China) and a microplate luminometer (BioTek, United States). Relative promoter activity was calculated by normalizing firefly luciferase activity to Renilla luciferase activity (Firefly/Renilla ratio) to control for transfection efficiency.

### 2.10 Measurement of intracellular free zinc

Intracellular free zinc levels were measured using the zinc-sensitive fluorescent probe FluoZin-3 AM (Abcam, United States). PASMCs were incubated with 2 μM FluoZin-3 AM in HEPES-buffered saline solution (HBSS; 140 mM NaCl, 5 mM KCl, 1 mM MgCl_2_, 2 mM CaCl_2_, 10 mM glucose, 10 mM HEPES, pH 7.4) supplemented with 0.02% Pluronic F-127 for 30 min at 37 ℃ in the dark. After loading, cells were washed twice with HBSS and incubated for an additional 20 min to allow de-esterification of the AM ester groups.

To verify probe specificity and enable quantitative comparison, calibration experiments were performed. For the minimum fluorescence (R_min_), cells were treated with the zinc chelator TPEN (50 μM, 10 min). For the maximum fluorescence (R_max_), cells were incubated with ZnSO_4_ (10 μM) plus pyrithione (2 μM) as an ionophore. Intracellular zinc concentrations were then estimated using the ratio method: *[Zn^2+^] = K_d_ × (F - F_min_) / (F_max_ - F)*, with K_d_ = 15 nM for FluoZin-3.

Confocal imaging was performed on a Zeiss LSM880 with a 40× oil objective, 488 nm excitation/500-550 nm emission, and identical laser power, gain, and pinhole across groups. Z-stacks were collected at 0.5 μm steps (6-10 optical sections) and rendered as maximum-intensity projections for quantification. Analyses were performed on five randomly selected fields per sample, with at least 80-100 cells evaluated per condition. Background subtraction used cell-free regions; photobleaching was corrected (ImageJ “Bleach Correction”). Quantification (nuclear/cytoplasmic or whole-cell intensity) was performed in ImageJ 1.54f by blinded analysts.

### 2.11 Transcriptome analysis

Transcriptome data of rat lung tissues (accession number: GSE186996) and human lung tissues (accession number: GSE15197 and GSE113439) were obtained from the Gene Expression Omnibus (GEO) database (https://www.ncbi.nlm.nih.gov/geo/). The raw RNA sequencing files were downloaded and processed using a standardized bioinformatics pipeline. After quality control, samples were stratified into *Mtf-1^high^* and *Mtf-1^low^* groups based on normalized expression values of *Mtf-1*. Differentially expressed genes (DEGs) between the two groups were identified using the *limma* package (version 3.54.2) in R, with statistical significance determined by adjusted *P*-values.

Gene Ontology (GO) enrichment analysis for biological processes was performed using the *enrichGO()* function in the *clusterProfiler* package (version 4.8.1). Gene Set Enrichment Analysis (GSEA) was conducted using ranked gene lists via the *gseGO()* function. Pathways were considered significantly enriched if they exhibited a false discovery rate (FDR) <0.25 and a normalized enrichment score (NES) > 1.

To identify candidate kinases responsible for MTF-1 phosphorylation at Ser^304^, in silico predictions were performed using NetPhos 3.1 (http://www.cbs.dtu.dk/services/NetPhos/) and PhosphoSitePlus (https://www.phosphosite.org/). Phosphorylation probability scores and log_2_-transformed kinase-substrate scores were used for ranking.

Correlation analysis between *Plgf* expression and members of the *Prkc* gene family was performed using Pearson correlation coefficients. Heatmaps and scatterplots were generated using the *pheatmap* (version 1.0.12) and *ggplot2* (version 3.4.4) packages, respectively.

### 2.12 Cleavage under targets and tagmentation (CUT&Tag)

Nuclei were isolated from treated and control cells, and immobilized on concanavalin A-coated magnetic beads. Samples were incubated overnight at 4 °C with primary antibodies against the MTF-1, followed by a secondary antibody to tether pA-Tn5 transposase. Tagmentation was initiated by addition of magnesium ions, allowing simultaneous cleavage and adapter insertion at antibody-bound chromatin sites. After DNA purification, sequencing libraries were amplified by PCR and subjected to paired-end sequencing (Illumina platform). Raw reads were trimmed using Trim Galore and aligned to the Rattus norvegicus genome (mRatBN7.2) with Bowtie2 using parameters optimized for CUT&Tag. Peak calling was performed with MACS2 (q < 0.05, fold enrichment > 5), and peaks were annotated with ChIPseeker. Signal density profiles were generated by deepTools, and average signal plots centered on transcription start sites (TSS ± 2 kb) were used to assess enrichment. GO and GSEA analyses were conducted using the *clusterProfiler* R package. Visualization of peak distribution and genome browser tracks was performed using Integrative Genomics Viewer (IGV).

### 2.13 Statistical analysis

Data are presented as mean ± standard deviation (SD). Statistical comparisons among groups were conducted using one-way analysis of variance (ANOVA), followed by Tukey's multiple-comparison test. A two-sided *P* value of less than 0.05 was considered statistically significant.

## 3. Results

### 3.1 MTF-1 was upregulated in the lungs of Su/Hx-PH rats

**Figure 1:**
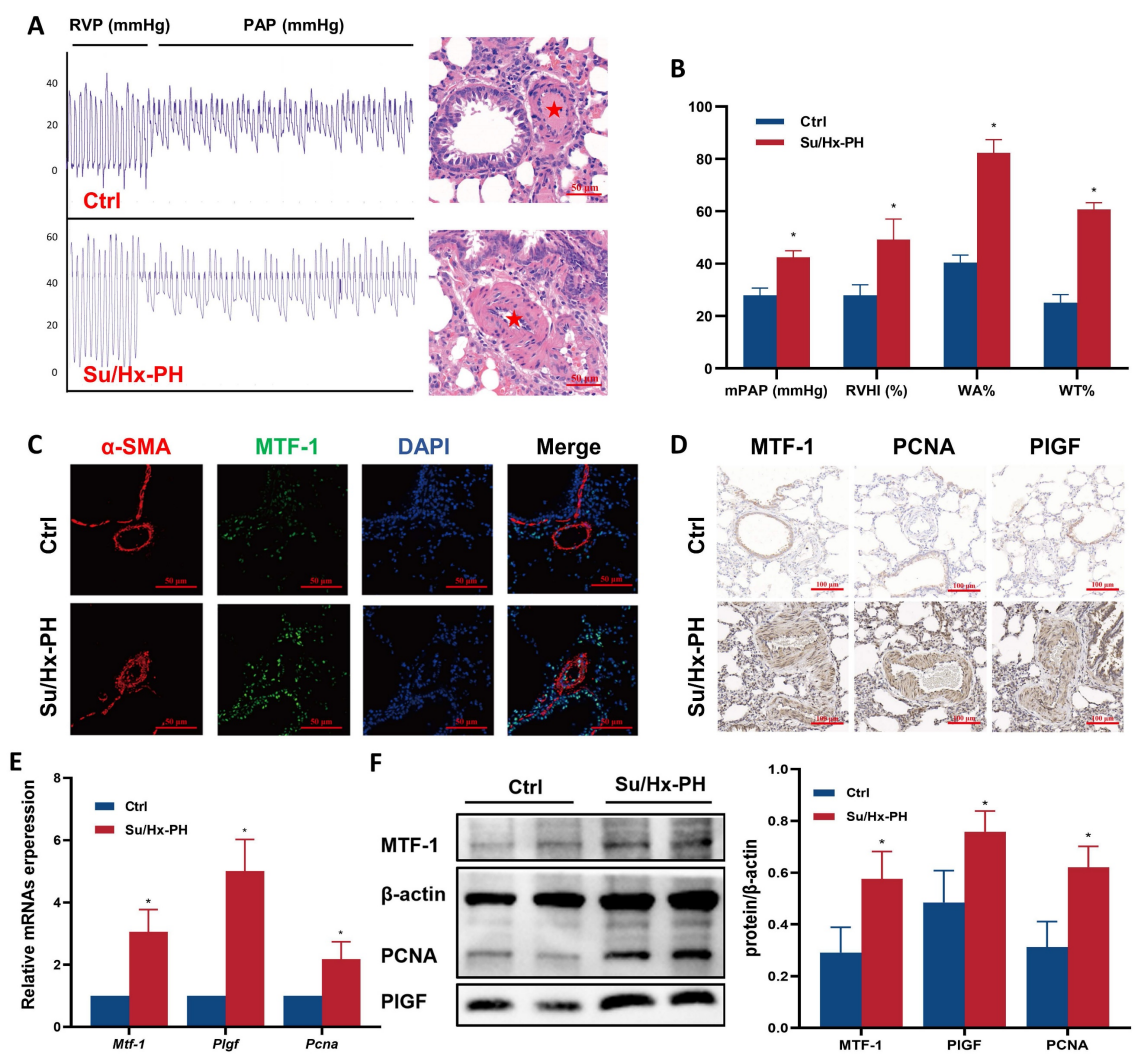
**Successful establishment of Su/Hx-PH rat model. (A)** Representative pulmonary arterial pressure tracings (left) and corresponding H&E lung sections (right) in Su/Hx-PH rats. The red asterisks indicated marked medial thickening and luminal narrowing of small pulmonary arteries. Scale bar=50 μm. **(B)** Elevated mPAP, RVHI, WA% and WT% in Su/Hx-PH group. **(C)** Immunofluorescence staining of MTF-1 and α-SMA in lung tissue. Scale bar=50 μm. **(D)** Immunohistochemical staining of MTF-1, PlGF, and PCNA. Scale bar=100 μm. **(E)** The mRNA expression levels of MTF-1, PlGF, and PCNA in lung tissue. **(F)** The protein expression levels of MTF-1, PlGF, and PCNA. ^*^P <0.05 vs. Ctrl. Data are represented as mean ± SD, n=8. Ctrl: control; Su/Hx-PH: PH induced by Sugen 5416 and hypoxia; H&E: Hematoxylin and Eosin; mPAP: mean pulmonary arterial pressure; RVHI: right ventricular hypertrophy index; WA%: wall area percentage; WT%: wall thickness percentage; MTF-1: metal-responsive transcription factor 1; α-SMA: α-smooth muscle actin; PlGF: placental growth factor; PCNA: proliferating cell nuclear antigen.

To determine whether MTF-1 was involved in the pathogenesis of PH, we quantified its transcripts and protein in lung tissues from Su/Hx-PH rats. Su/Hx-PH rats developed severe pulmonary hypertension, as reflected by elevated mPAP, increased RVHI, and pronounced pulmonary vascular remodeling, including higher WA% and WT% (**Figure 1A, B**). Immunofluorescence and immunohistochemistry demonstrated markedly stronger MTF-1 staining in the pulmonary vasculature of Su/Hx-PH rats compared with controls (**Figure 1C, D**). Consistently, quantitative analyses showed significant increases in MTF-1 mRNA and protein levels in lung tissue (**Figure 1E, F**). Expression of the MTF-1 downstream effectors PlGF and PCNA, which are associated with angiogenesis and proliferation, was also significantly elevated.

To assess the translational relevance of these findings, human pulmonary transcriptomic datasets (GSE15197 and GSE113439) were analyzed. Both *MTF1* and *PGF* mRNA levels were significantly higher in lung tissues from patients with PH (**Supplementary Figure 1A**), consistent with observations in Su/Hx-PH rats. These results collectively demonstrated that MTF-1 was activated in PH and promoted angiogenic genes expressions.

### 3.2 MTF-1 knockdown suppressed hypoxia- and zinc-induced PASMCs proliferation and migration

**Figure 2:**
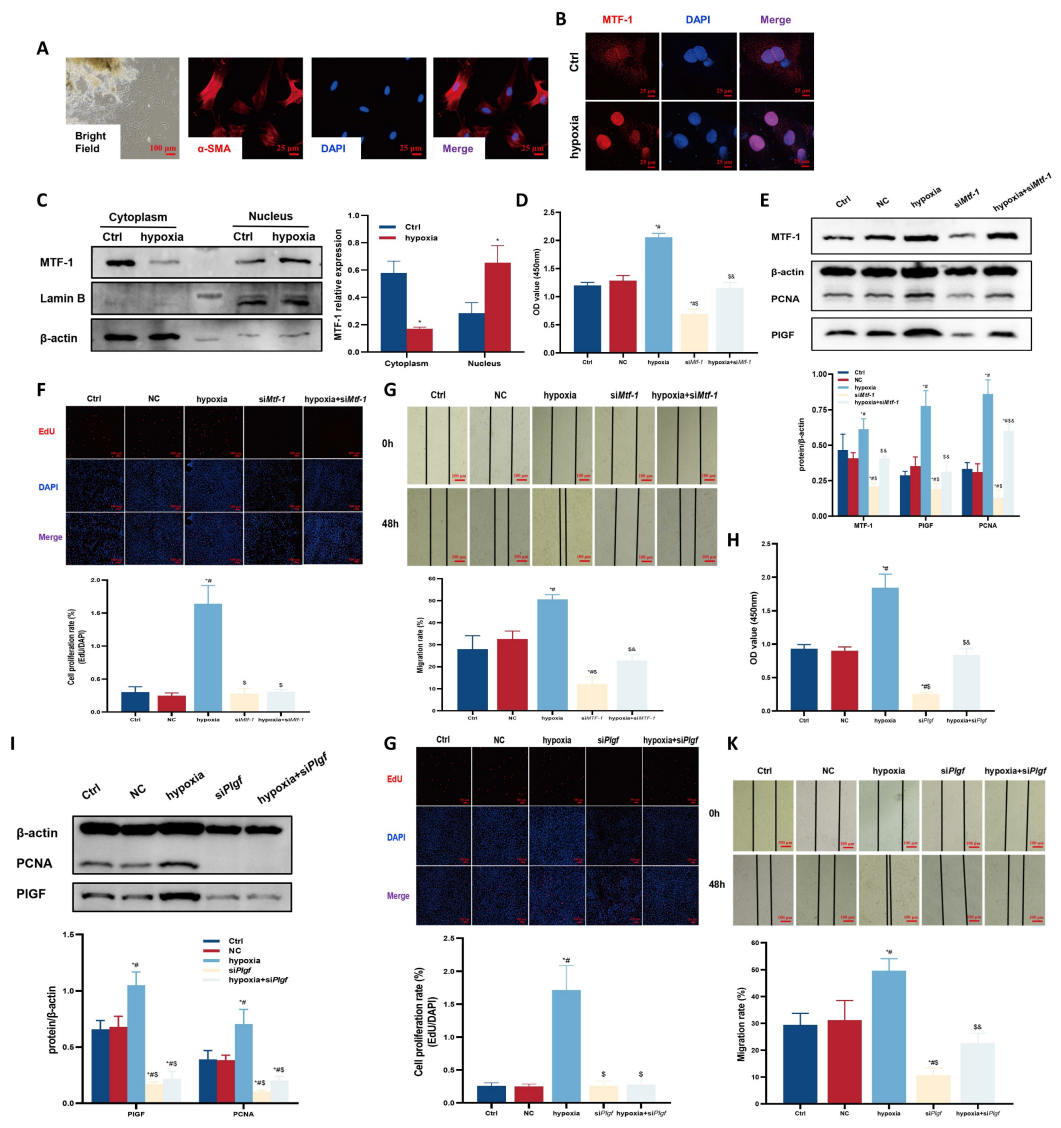
**Role of MTF-1 in hypoxia-induced proliferation and migration of PASMCs. (A)** Morphology and immunocytochemical identification of PASMCs. Bright field: scale bar=100 μm; immunofluorescence: scale bar=25 μm. **(B)** Immunocytochemistry showing hypoxia-induced nucleocytoplasmic translocation of MTF-1. Scale bar=25 μm. **(C)** Western blot analysis of MTF-1 translocation under hypoxia. **(D-G)** Effects of *Mtf-1* knockdown on PASMCs proliferation detected by CCK-8 assay (D), Western blot (E), EdU assay (F), and scratch assay (G). **(H-K)** Effects of *Plgf* knockdown on PASMCs proliferation detected by CCK-8 assay (H), Western blot (I), EdU assay (J), and scratch assay (K). ^*^P <0.05 vs. Ctrl; ^#^*P* <0.05 vs. NC; ^$^*P* <0.05 vs. hypoxia; ^&^*P* <0.05 vs. si*Mtf-1* or si*Plgf*. EdU and scratch assay: scale bar=100 μm. Data are represented as mean ± SD, n=5. Ctrl: control; NC: negative control; MTF-1: metal-responsive transcription factor 1; *Mtf-1*: encoding metal-responsive transcription factor 1 (Rat); *Plgf*: encoding placental growth factor (Rat); PASMCs: pulmonary arterial smooth muscle cells; CCK-8: Cell Counting Kit-8; EdU: 5-ethynyl-2'-deoxyuridine.

To confirm the role of MTF-1 in PASMCs proliferation and migration under hypoxic and zinc-excessive conditions, MTF-1 expression was silenced in primary PASMCs. The cells displayed a characteristic “hill-and-valley” morphology, and α-smooth muscle actin (α-SMA) immunofluorescence was used to confirm their identity (**Figure 2A**). Under hypoxic conditions, MTF-1 was translocated from the cytoplasm to the nucleus (**Figure 2B, C**), accompanied by increased expression of MTF-1 and PlGF.

To further explore how MTF-1 regulated transcriptional programs under hypoxic conditions, dual-luciferase reporter assay and CUT&Tag analysis were performed to map its chromatin binding landscape. Dual-luciferase reporter assays showed that wild-type MTF-1 activated the *Plgf* promoter in an MRE-dependent manner under both hypoxic and zinc-enriched conditions (**Supplementary Figure 1B**). CUT&Tag analysis revealed a prominent enrichment of MTF-1 binding near TSS (**Supplementary Figure 1C**), with the majority of peaks located in promoter regions (**Supplementary Figure 1D**). Comparative analysis between normoxia and hypoxia-treated samples identified extensive hypoxia-specific chromatin remodeling (**Supplementary Figure 1E**), and GO as well as GSEA analyses of these peaks highlighted biological processes related to vascular development and angiogenesis (**Supplementary Figure 1F-G**). Notably, a robust increase in chromatin occupancy was detected at the *Plgf* locus under hypoxia (**Supplementary Figure 1H**), indicating that PlGF was a direct transcriptional target of MTF-1.

Hypoxia-induced proliferation and migration were also diminished following MTF-1 silencing, as evidenced by decreased PCNA expression, reduced EdU incorporation, and impaired scratch wound closure (**Figure 2D-G**). Similar suppressive effects were observed upon PlGF knockdown (**Figure 2H-K**).

**Figure 3:**
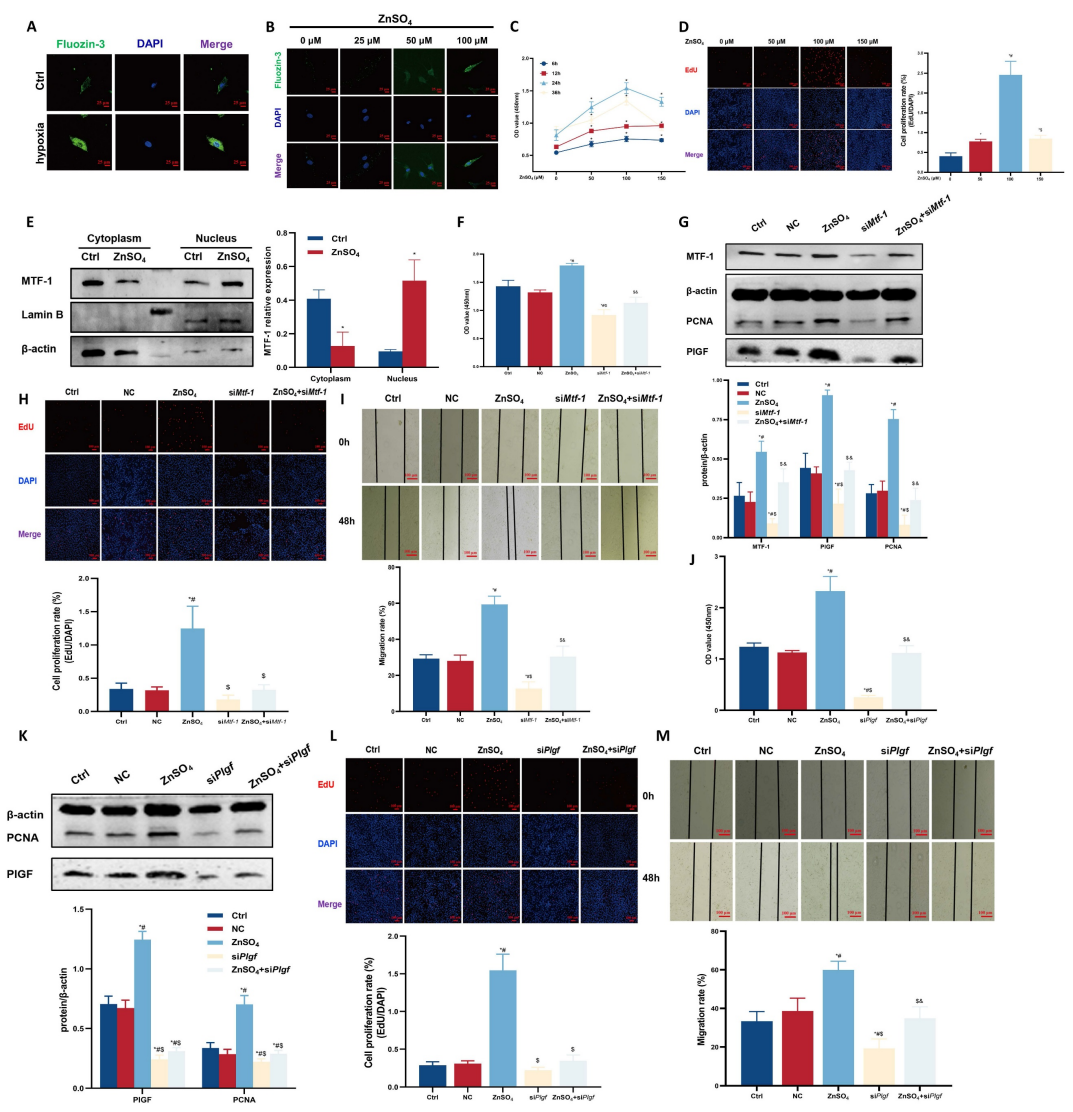
**Role of MTF-1 in ZnSO_4_-induced proliferation and migration of PASMCs. (A-B)** Intracellular zinc levels in hypoxic (A) or ZnSO_4_-treated (B) PASMCs detected by FluoZin-3 staining. Scale bar=25 μm. **(C-D)** Effects of ZnSO_4_ on proliferation of PASMCs detected by CCK-8 assay (C) and EdU assay (D). **(E)** Western blot analysis of MTF-1 translocation in response to ZnSO_4_. **(F-I)** Effects of *Mtf-1* knockdown on ZnSO_4_-treated PASMCs proliferation detected by CCK-8 assay (F), Western blot (G), EdU assay (H), and scratch assay (I). **(J-M)** Effects of *Plgf* knockdown on ZnSO_4_-treated PASMCs proliferation detected by CCK-8 assay (J), Western blot (K), EdU assay (L), and scratch assay (M). ^*^P <0.05 vs. Ctrl; ^#^*P* <0.05 vs. NC; ^$^*P* <0.05 vs. ZnSO_4_; ^&^*P* <0.05 vs. si*Mtf-1* or si*Plgf*. EdU and scratch assay: scale bar=100 μm. Data are represented as mean ± SD, n=5. Ctrl: control; NC: negative control; ZnSO_4_: zinc sulfate; MTF-1: metal-responsive transcription factor 1; PASMCs: pulmonary arterial smooth muscle cells; *Mtf-1*: encoding metal-responsive transcription factor 1 (Rat); *Plgf*: encoding placental growth factor (Rat); CCK-8: Cell Counting Kit-8; EdU: 5-ethynyl-2'-deoxyuridine.

Hypoxia and ZnSO_4_ treatment led to elevated intracellular free zinc levels (**Figure 3A, B**). Dose-response assays (25-200 μM ZnSO_4_) demonstrated that 100 μM for 24 h produced the most pronounced proliferative effect without detectable cytotoxicity, and this concentration was therefore used for subsequent experiments (**Figure 3C, D**). This treatment induced nuclear accumulation of MTF-1 (**Figure 3E**) and promoted PASMCs proliferation and migration, both of which were attenuated by knockdown of either MTF-1 (**Figure 3F-I**) or PlGF (**Figure 3J-M**).

Together, these results demonstrated that MTF-1 played an essential role in mediating PASMCs proliferation and migration in response to hypoxia and zinc, in part via upregulation of PlGF.

### 3.3 Phosphorylation of MTF-1 at Serine 304 (Ser^304^) was required for PASMCs proliferation and migration

**Figure 4:**
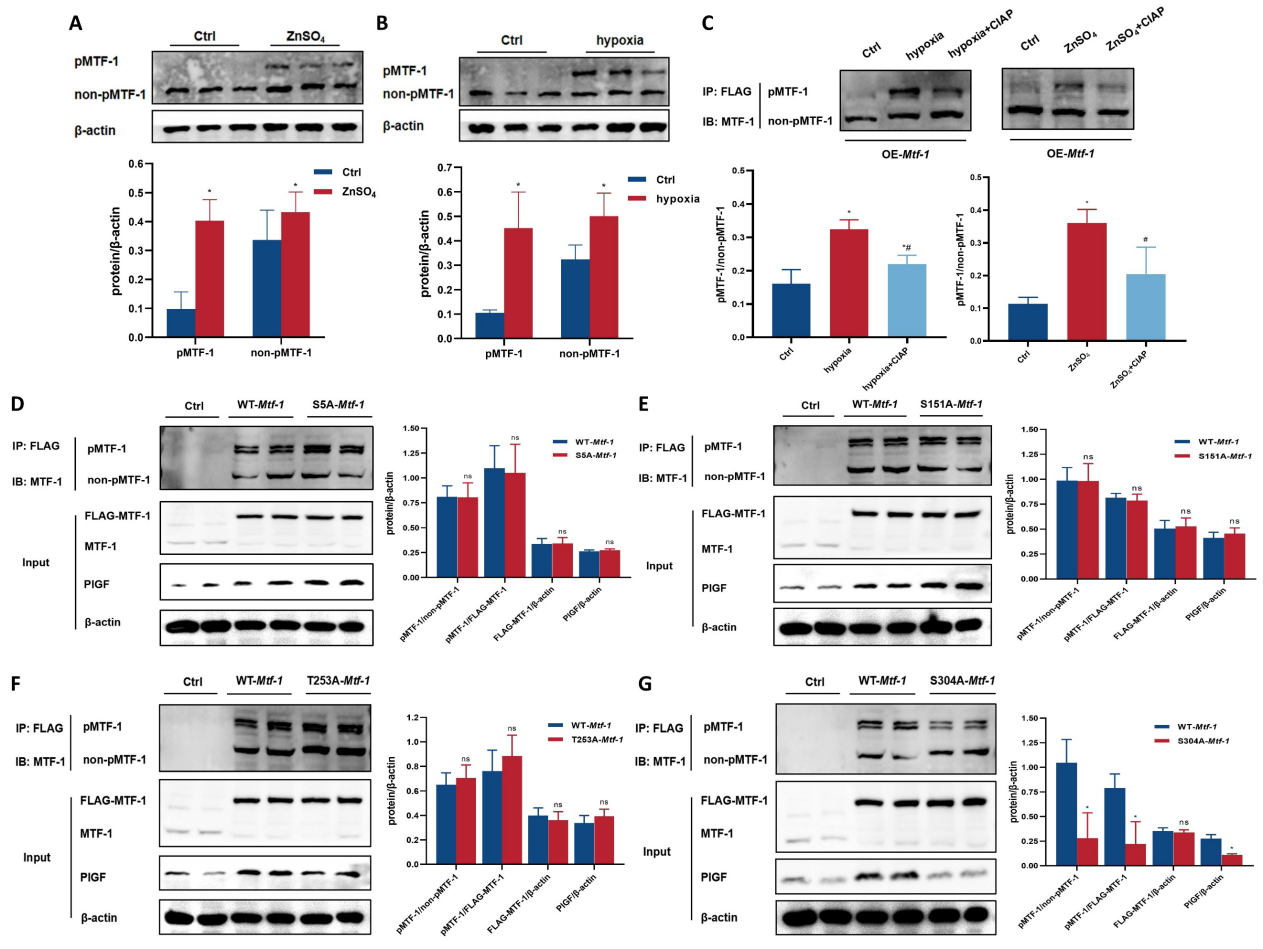
**Role of MTF-1 phosphorylation in proliferation and migration of PASMCs. (A-B)** Detection of MTF-1 phosphorylation in hypoxia- (A) and ZnSO_4_-treated (B) PASMCs using Phos-tag SDS-PAGE. **(C)** Verification of phosphorylation specificity by CIAP treatment (1 U/µg protein). **(D-G)** Effect of S5A-*Mtf-1* (D), S151A-*Mtf-1* (E), T253A-*Mtf-1* (F), and S304A-*Mtf-1* (G) overexpression on MTF-1 phosphorylation. ^*^P <0.05 vs. Ctrl. Data are represented as mean ± SD, n=5. Ctrl: control; ZnSO_4_: zinc sulfate; MTF-1: metal-responsive transcription factor 1; PASMCs: pulmonary arterial smooth muscle cells; Phos-tag: Phosphate-binding tag; SDS-PAGE: Sodium Dodecyl Sulfate Polyacrylamide Gel Electrophoresis; CIAP: calf intestinal alkaline phosphatase; *Mtf-1*: encoding metal-responsive transcription factor 1 (Rat).

To investigate the functional relevance of MTF-1 phosphorylation in PASMCs, site-directed mutagenesis was performed to generate alanine substitutions at candidate phosphorylation residues (S5A, S151A, T253A, and S304A). Under hypoxic or zinc-enriched conditions, Phos-tag SDS-PAGE revealed a mobility-shifted band corresponding to phosphorylated MTF-1 (pMTF-1), which was eliminated following treatment with CIAP, confirming its phosphorylation-dependent nature (**Figure 4A-C**). Among all mutants, only the S304A substitution markedly reduced MTF-1 phosphorylation and suppressed PlGF expression under stimulatory conditions (**Figure 4D-G**). These findings established Ser^304^ as a functionally indispensable phosphorylation site for MTF-1 activation under hypoxia and zinc stimulation.

### 3.4 PKC mediated Ser304 phosphorylation and activation of MTF-1

**Figure 5:**
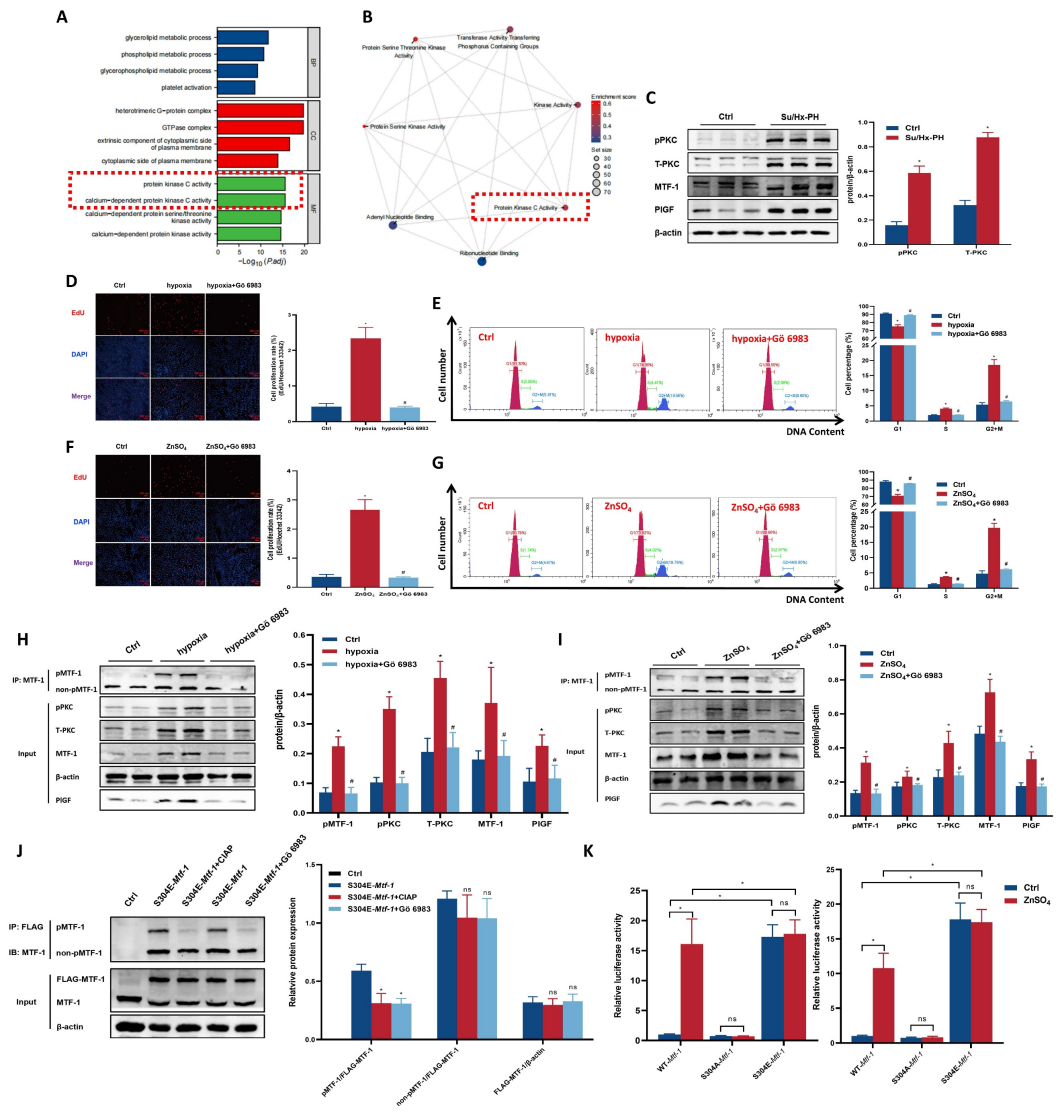
**Role of PKC kinase in MTF-1 phosphorylation and transcriptional activity. (A)** GO enrichment analysis of DEGs between *Mtf-1*^high^ and *Mtf-1*^low^ expression groups in GSE186996. **(B)** GSEA of DEGs between *Mtf-1*^high^ and *Mtf-1*^low^ expression groups in GSE186996. **(C)** Western blot showing phosphorylated and total PKC levels in lung tissues from Su/Hx-PH rats. **(D-I)** Effect of Gö 6983 on proliferation of hypoxia- (D, E, H) or ZnSO_4_-treated (F, G, I) PASMCs detected by EdU assay, flow cytometry, and Western blot. EdU assay: scale bar=100 μm. **(J)** Effect of CIAP and Gö 6983 on pMTF-1 expression. **(K)** Luciferase reporter assay evaluating the effect of Ser^304^ phosphorylation on MTF-1 transcriptional activity in HEK293T cells. ^*^P <0.05 vs. Ctrl; ^#^*P* <0.05 vs. hypoxia or ZnSO_4_. Data are represented as mean ± SD, n=5. Ctrl: control; Su/Hx-PH: PH induced by Sugen 5416 and hypoxia; ZnSO_4_: zinc sulfate; hypoxia+Gö 6983: hypoxic PASMCs+10 μM Gö 6983. ZnSO_4_+Gö 6983: ZnSO_4_-treated PASMCs+10 μM Gö 6983; PKC: protein kinase C; MTF-1: metal-responsive transcription factor 1; GO: Gene Ontology; DEGs: differentially expressed genes; GSEA: Gene Set Enrichment Analysis; *Mtf-1*: encoding metal-responsive transcription factor 1 (Rat); EdU: 5-ethynyl-2'-deoxyuridine; CIAP: calf intestinal alkaline phosphatase.

To further elucidate the biological pathways associated with MTF-1 activation, we stratified samples from GSE186996 into *Mtf-1*^high^ and *Mtf-1*^low^ expression groups and performed transcriptome-wide analysis. GO enrichment of DEGs revealed significant enrichment in biological processes related to protein kinase C signaling and phosphorylation (**Figure 5A**). GSEA further demonstrated positive enrichment of hallmark pathways involving PKC activity in the *Mtf-1*^high^ group (**Figure 5B**).

To identify the specific kinase responsible for Ser^304^ phosphorylation of MTF-1, we next conducted in silico kinase prediction analyses. NetPhos 3.1 identified Ser^304^ as a high-confidence phosphorylation site, with PKC ranked among the top candidate kinases (score =0.810, **Supplementary Table 4**). Complementary predictions from PhosphoSitePlus highlighted multiple PKC isoforms—particularly particularly PKCθ (*Prkcq*), PKCγ (*Prkcg*), PKCβ (*Prkcb*), and PKCδ (*Prkcd*)—as likely upstream kinases (**Supplementary Table 5**), and correlation analysis of transcriptomic data showed a strong positive correlation between *Plgf* and all *Prkc* isoforms expressions (**Supplementary Figure 2A**). Furthermore, correlation analysis of transcriptomic data revealed that *Plgf* expression exhibited the strongest positive correlation with *Prkcd* (r =0.94, **Supplementary Figure 2B**). Taken together, these convergent lines of evidence strongly implicated PKCδ as the most plausible upstream kinase mediating Ser^304^ phosphorylation of MTF-1.

Experimentally, PASMCs were treated with the pan-PKC inhibitor Gö 6983 (10 μM). Pulmonary tissues from hypoxic rats exhibited elevated both total and phosphorylated PKC (**Figure 5C**). Gö 6983 treatment inhibited hypoxia- and ZnSO_4_-induced PASMCs proliferation, abolished MTF-1 Ser^304^ phosphorylation, and reduced *Plgf* transcriptional activity (**Figure 5D-K**). Collectively, these results established PKC-dependent phosphorylation of MTF-1 at Ser^304^ as a prerequisite for its transcriptional and pro-proliferative functions. To further verify the specific role of PKCδ in mediating MTF-1 phosphorylation, PASMCs were transfected with si*Prkcd* to knock down PKCδ expression. Similar inhibitory effects were observed following si*Prkcd* transfection in ZnSO_4_-treated PASMCs (**Supplementary Figure 2C, D**), in which a marked reduction of pPKCδ and pMTF-1 levels were evident by Western blot analysis (**Supplementary Figure 2E**).

### 3.5 Pharmacological inhibition of MTF-1 by APTO-253 attenuated hypoxia-induced pulmonary vascular remodeling

**Figure 6:**
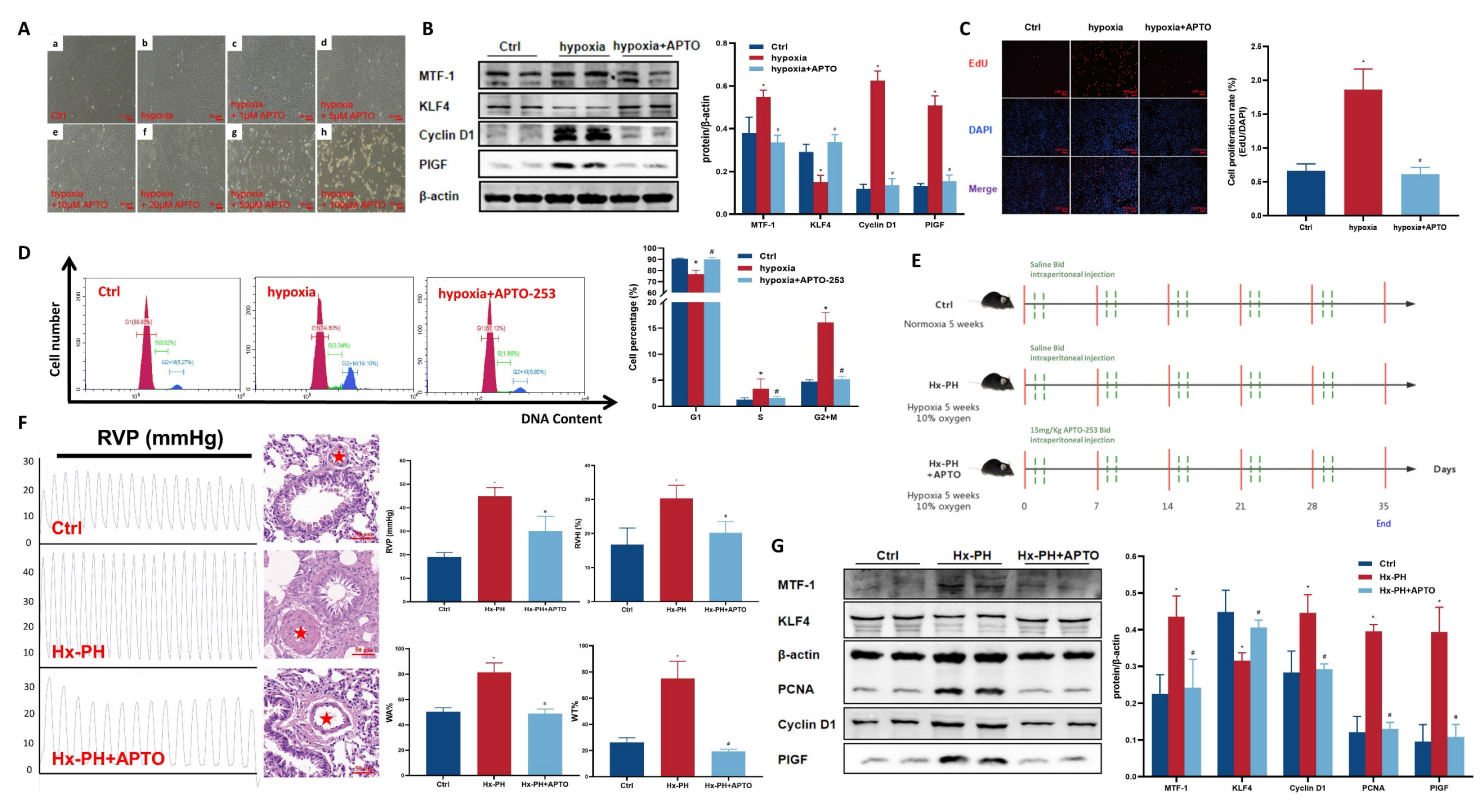
** Effect of APTO-253 on hypoxia-induced PH. (A)** Morphological changes of hypoxic PASMCs treated with varying concentrations of APTO-253. The red asterisks indicated marked medial thickening and luminal narrowing of small pulmonary arteries. Scale bar=50 μm. **(B-D)** Effect of APTO-253 on protein expression proliferation, and cell cycle in hypoxic PASMCs, assessed by Western blot (B), EdU assay (C), and flow cytometry (D). EdU assay: scale bar=100 μm. **(E)** Schematic of intraperitoneal APTO-253 administration in mice. Mice received APTO-253 (15 mg/kg, intraperitoneal injection twice weekly) during the 5-week hypoxic exposure. **(F)** Effect of APTO-253 on RVP and pulmonary vascular remodeling in Hx-PH mice. Hemodynamic measurements were obtained by closed-chest transthoracic intercostal puncture using a PowerLab recording system. Scale bar=50 μm. **(G)** Effect of APTO-253 on protein expression in lung tissues of Hx-PH mice. Cell experiments: ^*^P <0.05 vs. Ctrl; ^#^*P* <0.05 vs. Hypoxia. Data are represented as mean ± SD, n=5. Ctrl: control; hypoxia+APTO: hypoxic PASMCs+10 μM APTO-253; PASMCs: pulmonary arterial smooth muscle cells; EdU: 5-ethynyl-2'-deoxyuridine. Animal experiments: ^*^P <0.05 vs. Ctrl; ^#^*P* <0.05 vs. Hx-PH. Data are represented as mean ± SD, n=8. Ctrl: control; Hx-PH: hypoxia induced PH; Hx-PH+APTO: Hx-PH with APTO-253 treatment; RVP: right ventricular pressure.

To verify the therapeutic potential of targeting MTF-1 in PH, APTO-253, a selective MTF-1 inhibitor, was administered both *in vitro* and *in vivo*. Microscopic dose-response observations showed that APTO-253 exerted a concentration-dependent inhibitory effect on PASMCs proliferation. Among the tested doses (1-100 μM), 10 μM produced the strongest anti-proliferative effect while maintaining normal cell morphology and viability, and was therefore selected for subsequent *in vitro* studies (**Figure 6A**). Under hypoxic conditions, APTO-253 treatment reduced the expression of cell cycle-associated proteins, including PCNA and cyclin D1, and suppressed EdU incorporation (**Figure 6B-D**).

*In vivo*, mice subjected to chronic hypoxia received APTO-253 (**Figure 6E**). Treatment markedly reduced medial wall thickness of distal pulmonary arteries, as quantified by α-SMA staining, and hemodynamic measurements showed significant reductions in RVSP and RVHI compared to vehicle-treated controls (**Figure 6F**). Moreover, APTO-253 suppressed hypoxia-induced upregulation of MTF-1 and its downstream targets KLF4, PCNA, Cyclin D1, and PlGF in lung tissues (**Figure 6G**).

Collectively, these findings demonstrated that pharmacological blockade of MTF-1 by APTO-253 mitigated hypoxia-driven pulmonary vascular remodeling and improved cardiopulmonary hemodynamics, highlighting MTF-1 as a pathogenic driver and potential therapeutic target in PH.

## 4. Discussion

**Figure 7:**
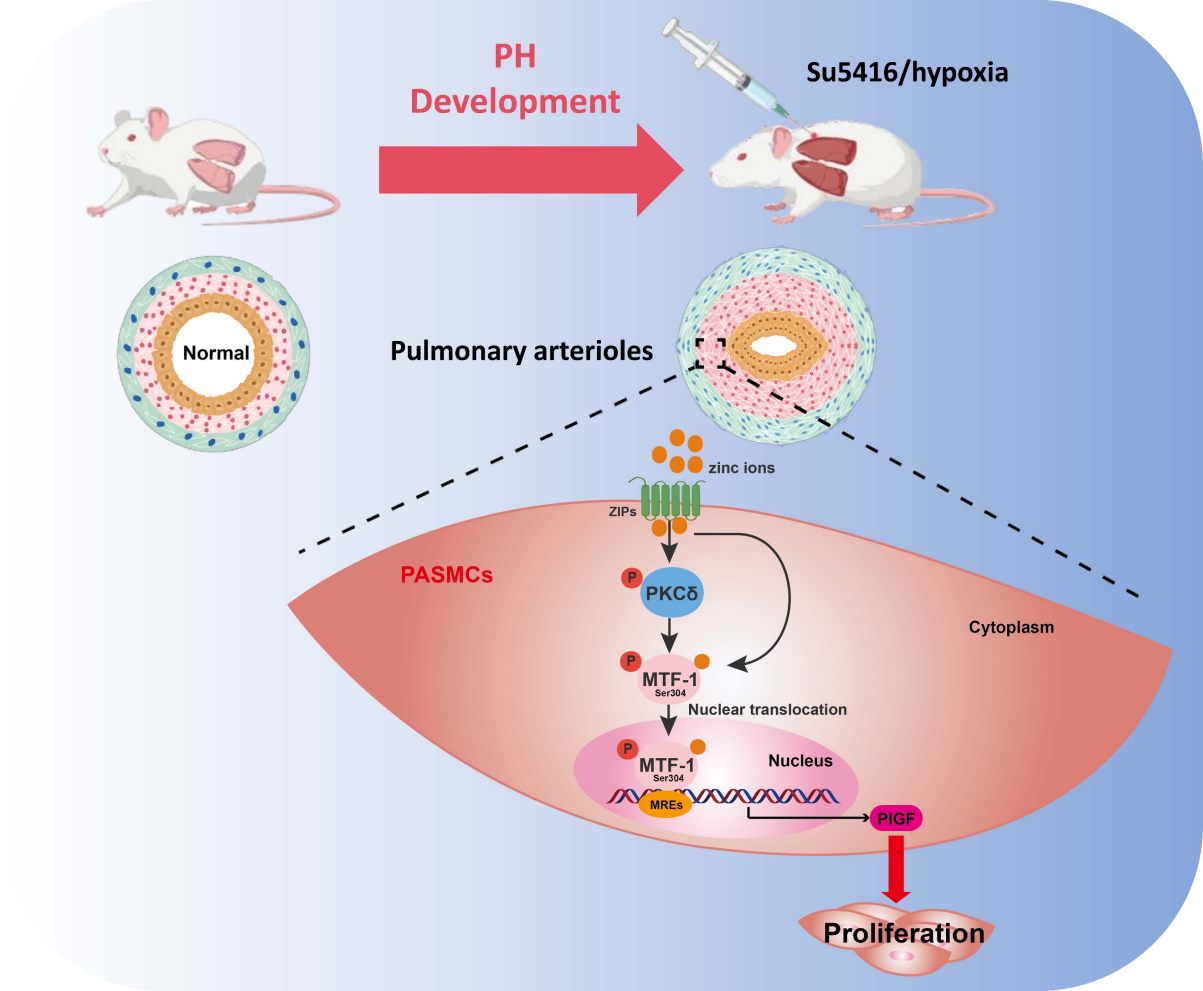
**Schematic illustration of the role and regulatory mechanism of MTF-1 in PASMCs proliferation and migration.** Zinc ions enter cells via ZIP transporters, activate PKCδ, and promote MTF-1 phosphorylation and nuclear translocation. Phosphorylated MTF-1 binds to MREs to upregulate PlGF, enhancing PASMCs proliferation and migration. ZIPs: Zrt- and Irt-like proteins; PKCδ: protein kinase Cδ; MTF-1: metal-regulatory transcription factor 1; MREs: metal response elements; PlGF: placental growth factor; PASMCs: pulmonary arterial smooth muscle cells; PH: pulmonary hypertension.

In this study, we systematically investigated the role of MTF-1 in the pathogenesis of hypoxia-induced PH. We found that MTF-1 expression and transcriptional activity were markedly upregulated under hypoxic and zinc-enriched conditions, both *in vitro* and *in vivo*. Pharmacological inhibition of MTF-1 using APTO-253 significantly reduced pulmonary arteriolar remodeling and attenuated the PASMCs proliferation, underscoring the therapeutic relevance of targeting MTF-1 in PH. Mechanistically, we identified serine 304 (Ser^304^) as a critical phosphorylation site essential for MTF-1 activation. Site-directed mutagenesis revealed that phosphorylation at Ser^304^ was necessary for the pro-proliferative and pro-migratory functions of MTF-1 in PASMCs. Furthermore, we showed that the PKC inhibitor Gö 6983 suppressed PASMCs proliferation, at least in part, by inhibiting MTF-1 phosphorylation and downregulating the expression of its downstream effector, PlGF. Consistently, specific knockdown of PKCδ reproduced these effects, confirming PKCδ as the predominant isoform mediating MTF-1 activation. Collectively, our data supported a model in which PKCδ-mediated phosphorylation of MTF-1 at Ser^304^ enhanced its transcriptional activity, leading to PlGF induction and pathological PASMCs remodeling (**Figure 7**). These findings defined a previously uncharacterized PKCδ/MTF-1/PlGF axis that contributed to hypoxia-driven pulmonary vascular remodeling and highlighted MTF-1 as a potential molecular target for therapeutic intervention in PH.

Our results established MTF-1 as a transcriptional hub linking hypoxia to aberrant vascular smooth muscle proliferation. Consistent with its known role in maintaining metal ion and oxidative balance^20^, ^21^, MTF-1 was significantly upregulated in hypoxic PH models and localized to pulmonary vascular tissues. Functional analyses revealed that MTF-1 knockdown markedly suppressed hypoxia- and zinc-induced PASMCs proliferation and migration, while its downstream targets PlGF and PCNA were elevated in diseased lungs. These findings extend prior observations in monocrotaline (MCT)-induced PH and underscore MTF-1 as a nodal effector of proliferative and angiogenic responses in pulmonary arteries. Importantly, although our data supported a pathogenic role, MTF-1 might exert context-dependent protective effects, particularly in early disease stages, by buffering oxidative stress or preserving zinc homeostasis. Such duality, also observed in cancer biology, highlights the need to dissect temporal and cell-type-specific functions of MTF-1 in PH^22^, ^23^. In addition to PASMCs, other vascular cell types, including endothelial cells and fibroblasts, may also be subject to MTF-1-dependent regulation in hypoxia. Endothelial dysfunction is a key initiator of PH, driving vasoconstriction, microthrombosis, and aberrant angiogenesis^24^-^26^, while fibroblast activation contributes to adventitial thickening and extracellular matrix remodeling^27^, ^28^. Although we did not directly investigate these cell types, it is plausible that MTF-1 activation in endothelial cells could amplify inflammatory and angiogenic responses, and that its induction in fibroblasts might promote fibrotic remodeling. Thus, our conclusions should be interpreted primarily in the context of PASMCs biology. Future studies applying single-cell transcriptomics, spatial omics, or conditional knockout models will be essential to clarify whether MTF-1 exerts cell-type-specific functions across the vascular wall and to define its multicellular contribution to PH pathogenesis.

Beyond the hypoxia-induced and Su/Hx-PH models used in this study, several other experimental paradigms have been widely employed to investigate pulmonary hypertension, including MCT-induced PH and chronic or intermittent hypobaric hypoxia^29^. Each model recapitulates distinct aspects of PH pathophysiology and has inherent advantages and limitations. The MCT model produces severe pulmonary vascular remodeling and right ventricular hypertrophy through endothelial injury and inflammation^30^, but its pathology is driven by a toxic metabolite that does not naturally occur in human disease, limiting its direct clinical relevance. Intermittent hypobaric hypoxia, often used to mimic obstructive sleep apnea, emphasizes oscillatory hypoxia-reoxygenation injury but does not fully reproduce the progressive vascular remodeling of chronic PH^31^. By contrast, the chronic hypoxia and Su/Hx models capture sustained hypoxic vasoconstriction and proliferative vascular remodeling, which are core features of human chronic hypoxic PH^32^, ^33^. Thus, while our findings convincingly established the zinc-PKCδ-MTF-1-PlGF axis in hypoxia-driven PH, extrapolation to inflammation-dominant or toxin-induced forms of PH should be made with caution. Acknowledging these differences clarifies both the strength of our model in reproducing the pathophysiology of chronic hypoxia-related PH in humans and the need for further validation in complementary models that emphasize alternative disease mechanisms.

Hypoxia triggered a rapid elevation in intracellular free zinc, which facilitated MTF-1 nuclear translocation and transcriptional activation. Zinc, as an essential trace element, exerts biphasic effects on vascular smooth muscle cells: low concentrations (<80 μM) promote proliferation, whereas high concentrations (>100 μM) may induce cytotoxicity depending on lineage and buffering capacity^34^. Our results showed that 100 μM ZnSO_4_ significantly enhanced PASMCs proliferation via PKCδ-dependent activation of MTF-1 and PlGF. These findings suggest that tissue- or lineage-specific zinc sensitivity, coupled with differential expression of zinc transporters or PKC isoforms, may explain discrepancies across vascular beds. For instance, coronary artery smooth muscle cells exhibit greater susceptibility to zinc-induced apoptosis than PASMCs^35^, reflecting variations in zinc handling. Moreover, zinc signaling may also interact with alternative kinase pathways, and potentially engage in feedback loops that regulate MTF-1 transcriptional activity. In vascular smooth muscle cells, zinc has been shown to activate multiple signaling cascades beyond PKC, including the PI3K/AKT, MAPK/ERK^5^, and JNK^36^ pathways, which regulate cellular proliferation, survival, and phenotypic switching. These alternative zinc-responsive pathways may intersect with MTF-1 signaling or function in parallel to coordinate PASMCs responses under hypoxic or inflammatory conditions. Collectively, these results position zinc homeostasis as a central modulator of hypoxia-driven remodeling, reinforcing the necessity of cell type-specific investigation in vascular pathobiology.

A major advance of this study was the identification of Ser^304^ phosphorylation as indispensable for MTF-1 activity in PASMCs. Using site-directed mutagenesis and Phos-tag SDS-PAGE, we demonstrated that phosphorylation at Ser^304^ was both necessary and sufficient for PlGF transcriptional activation, independent of nuclear trafficking. Functionally, Ser^304^ substitution suppressed proliferation and migration under hypoxic and zinc-enriched conditions. Comparative analyses further highlight the remarkable diversity of MTF-1 phosphoregulation: CK2 mediates nuclear retention during heavy metal exposure^37^, ERK modulates oxidative stress responses^38^, and Ser^60^ phosphorylation governs cadmium responses^39^, whereas PP2A dephosphorylation^40^ and zinc depletion^41^ counteract activation. The evolutionary conservation of Ser^304^ suggested a specialized role in vertebrate hypoxia adaptation. Future work should explore how Ser^304^ phosphorylation intersects with other post-translational modifications and whether this contributes to maladaptive vascular remodeling in PH.

Transcriptomic profiling and kinase prediction analyses identified PKC as upstream mediators of Ser^304^ phosphorylation. Pharmacological inhibition with Gö 6983 completely abolished Ser^304^ phosphorylation, PlGF induction, and PASMCs proliferation under hypoxia, confirming PKC as the nodal kinase in zinc/MTF-1 signaling. These results placed PKC within the broader signaling architecture of PH, where PKC isoforms regulated vascular tone, inflammation, and smooth muscle plasticity^42^. Importantly, PKC represents a large kinase family with distinct isoforms exerting nonredundant functions in vascular biology. For example, PKCδ has been linked to pro-apoptotic and stress-responsive pathways^43^, ^44^, whereas PKCε often promotes cell survival and adaptive remodeling^45^, ^46^. The observation that multiple PKC isoforms emerged as candidate kinases for Ser^304^ suggested that MTF-1 phosphorylation might integrate signals from different PKC subfamilies. This raises the critical question of therapeutic selectivity, as pan-PKC inhibitors effectively suppress Ser^304^ phosphorylation but at the same time interfere with other PKC-mediated physiological processes, potentially leading to systemic toxicity. In contrast, selectively targeting disease-relevant isoforms may preserve protective PKC functions while attenuating pathogenic signaling, thereby offering a more refined and safer therapeutic strategy.

Correlation analysis of transcriptomic data further revealed that *Plgf* expression exhibited the strongest positive correlation with Prkcd, suggesting PKCδ as a likely upstream regulator. To experimentally validate this association, PASMCs were transfected with si*Prkcd* to selectively knock down PKCδ expression. PKCδ silencing recapitulated the effects of pan-PKC inhibition, resulting in markedly reduced phosphorylation of both PKCδ and MTF-1 and confirming PKCδ as the predominant isoform mediating MTF-1 activation under zinc stimulation. These results refined our mechanistic model by identifying a specific PKC isoform that linked zinc signaling to MTF-1 phosphorylation and downstream PlGF induction. Nonetheless, transcriptomic analyses indicated that multiple PKC isoforms, including PKCα and PKCβ, are expressed in pulmonary vascular tissues and may participate in distinct aspects of vascular remodeling, such as endothelial dysfunction and inflammation^47^, ^48^. Given the context-dependent and sometimes opposing roles of PKC isoforms, it is conceivable that different subfamilies cooperate or compete to shape the overall vascular phenotype. Selective targeting of PKCδ may therefore provide a more precise therapeutic strategy that interrupts pathogenic zinc/MTF-1 signaling while preserving other protective PKC functions. Future studies using isoform-specific inhibitors or genetic models will be critical to define the contributions of individual PKC isoforms *in vivo* and to determine whether isoform-selective inhibition can achieve efficacy with fewer adverse effects than broad PKC blockade.

The translational significance of our findings lied in the demonstration that APTO-253, originally developed as an anti-cancer agent targeting MTF-1 in acute myeloid leukemia^49^, ^50^, confers robust protection against vascular remodeling. APTO-253 suppressed PASMCs proliferation *in vitro* and attenuated hypoxia-induced pulmonary vascular remodeling and right ventricular hypertrophy *in vivo*. Mechanistically, APTO-253 inhibited MTF-1 transcriptional activity, leading to downregulation of Cyclin D1 and upregulation of KLF4, a cell-cycle suppressor implicated in PASMCs growth restraint. This pathway might partially explain the anti-proliferative effects observed. Prior studies supported a protective role of KLF4 in PH, where overexpression suppressed ERK1/2 signaling and inhibited hypoxia-induced proliferation and migration^51^. Conversely, aberrant KLF4 nuclear localization has been linked to mitochondrial dysfunction via the NDRG1/DRP1 axis, suggesting context-dependent functions^52^. Together, these findings indicated that APTO-253 not only directly targeted MTF-1 but also modulated downstream regulatory networks that converged on PASMCs proliferation.

In the context of current PH therapy, the significance of our findings becomes clearer. Existing agents, including endothelin receptor antagonists, nitric oxide pathway modulators, and prostacyclin analogs, primarily act as vasodilators^53^-^55^. They improve hemodynamics and quality of life, but have little effect on the proliferative and remodeling processes that drive disease progression, and patients frequently develop right heart failure despite treatment^56^, ^57^. This therapeutic gap underscores the need for disease-modifying approaches. Targeting the zinc-PKCδ-MTF-1-PlGF axis with APTO-253 directly suppresses PASMCs proliferation and vascular remodeling, addressing a central pathogenic mechanism that current therapies largely leave untreated. Moreover, the combination of MTF-1-based interventions with standard vasodilator therapies could provide complementary benefits. By simultaneously lowering pulmonary pressures and restraining vascular remodeling, this strategy has the potential to shift PH management from symptomatic palliation toward mechanism-directed therapy.

Importantly, the zinc-PKCδ-MTF-1-PlGF axis revealed here is not only a target for pharmacological intervention with APTO-253 but also a pathway with broader clinical implications. Altered zinc homeostasis has been reported in patients with chronic lung disease, pulmonary hypertension, and other cardiopulmonary disorders, suggesting that serum zinc levels or zinc transporter expression could serve as indicators of disease susceptibility or progression^4^, ^6^, ^9^. Likewise, PlGF has been recognized as a circulating marker of vascular remodeling and adverse cardiovascular outcomes, implying potential value for noninvasive monitoring of PH^58^-^60^. Beyond biomarker potential, this axis provides therapeutic opportunities: pharmacological inhibition of MTF-1, modulation of zinc transport, or blockade of PlGF signaling could complement current vasodilator-based treatments. Moreover, combined assessment of zinc status, PlGF levels, and MTF-1 activity may allow patient stratification into subgroups with distinct pathogenic drivers, thereby guiding personalized therapies. Future studies in clinical cohorts and prospective trials will be critical to validate these factors as biomarkers and to determine whether modulation of this pathway can be integrated into therapeutic strategies to improve long-term outcomes in PH.

Nevertheless, several limitations of this study should be acknowledged. First, although APTO-253 was effective in alleviating vascular remodeling in hypoxic mice, its long-term safety, pharmacokinetics, and potential efficacy in rats or humans remain undefined, which raises concerns about translational applicability. Second, while we demonstrated the involvement of the PKCδ/MTF-1/PlGF pathway in PASMCs, it remains possible that other pulmonary vascular cell types also contribute to MTF-1-mediated remodeling. Third, although our transcriptomic analysis of human PH lung tissues supported activation of the MTF-1/PlGF axis, the present findings were largely derived from rodent models and *in vitro* systems. Future studies using primary human PASMCs will be performed to validate MTF-1 activation and its downstream targets under hypoxic and zinc-enriched conditions, which will further strengthen the translational relevance of this pathway. Finally, although Ser^304^ was identified as a critical phosphorylation site, additional regulatory sites may exist and should be comprehensively explored through phosphoproteomic approaches.

In conclusion, this study uncovered a previously unrecognized role of MTF-1 phosphorylation in hypoxia-induced PH. Our data suggested that hypoxia elevated cytoplasmic free zinc, which in turn activated PKCδ and promoted MTF-1 phosphorylation at Ser^304^, leading to enhanced PlGF transcription and PASMCs proliferation. Targeting this zinc/PKCδ/MTF-1/PlGF signaling axis, either by restoring zinc homeostasis or pharmacologically inhibiting MTF-1, might offer a novel therapeutic strategy for PH. These findings broaden the understanding of zinc biology in vascular disease and provided new insights into the transcriptional control of PASMCs behavior under pathological hypoxia.

## Supplementary Material

Supplementary figures and tables.

## Abbreviations

ANOVA: one-way analysis of variance
α-SMA: α-smooth muscle actin
CCK-8: Cell Counting Kit-8
CIAP: calf intestinal alkaline phosphatase
CoCl_2_: cobalt chloride
co-IP: co-immunoprecipitation
DAPI: 4',6-diamidino-2-phenylindole
DEGs: differentially expressed genes
ECL: enhanced chemiluminescence
EdU: 5-ethynyl-2'-deoxyuridine
FDR: false discovery rate
GEO: Gene Expression Omnibus
GO: Gene Ontology
GSEA: Gene Set Enrichment Analysis
HBSS: HEPES-buffered saline solution
H&E: hematoxylin and eosin
IF: immunofluorescence
IHC: immunohistochemistry
MCT: monocrotaline
MREs: metal-responsive elements
MTF-1: metal-responsive transcription factor 1
NES: normalized enrichment score
PAP: pulmonary arterial pressure
PASMCs: pulmonary arterial smooth muscle cells
PH: Pulmonary hypertension
Phos-tag: phosphate-binding tag
PI: propidium iodide
PlGF: placental growth factor
RVHI: right ventricular hypertrophy index
RVP: right ventricular pressure
SD: standard deviation
SDS-PAGE: Sodium Dodecyl Sulfate Polyacrylamide Gel Electrophoresis
TSS: transcription start sites
WA%: wall area percentage
WT%: wall thickness percentage
ZnSO_4_: zinc sulfate
