# Supplementary material for: Zinc-Induced PKCδ-dependent Phosphorylation of MTF-1 Promotes Pulmonary Vascular Remodeling in Hypoxic Pulmonary Hypertension
Source: Int J Biol Sci. 2026 Jan 1;22(2):823–40. doi: 10.7150/ijbs.124664 (PMC12781076; doi:10.7150/ijbs.124664)
Supplement: Supplementary file 1 — Supplementary figures and tables. [file ijbsv22p0823s1.pdf]

Supplementary Materials

Supplementary Table 1 2

Supplementary Table 2 3

Supplementary Table 3 4

Supplementary Table 4 5

Supplementary Table 5 6

Supplementary Figure 1 8

Supplementary Figure 2 9

Supplementary Table 1 List of primers used in this study

**Genes**	**Sense (5' to 3')**	**Antisense (5' to 3')**
Mtf-1	5'-ACTCCTAACACGGCAATCCT-3'	5'-GTTCTGTGCTACTGGCTACTGGTA-3'
Plgf	5'-CCCTGTCTGCTGGGAACAAC-3'	5'-ACAGCGACTCAGAAGGACAC-3'
Pcna	5'-TGTCTCCTACAGTAACACTCAGT-3'	5'-ATCCTTCTTCATCTTCGATCTTGG-3'
Gapdh	5'-GGTTGTCTCCTGCGACTTCA-3'	5'-TGGTCCAGGGTTTCTTACTCC-3'

Supplementary Table 2 List of mutation sequences used in this study

Mutation site	Original sequence	Mutation sequence	State
S5A	MGEHSPDDN	MGEHAPDDN	Dephosphorylation
S151A	PRTYSTAGN	PRTYATAGN	
T253A	IRTHTGEKP	IRTHAGEKP	
S304A	STQYSLKSH	STQYALKSH	
S304E	STQYSLKSH	MGEHAPDDN	Continuous phosphorylation

Supplementary Table 3 List of siRNAs used in this study

siRNA	Sequence
Mtf-1-siRNA	5’-GCACAAUUUCACCAGCAAUTT-3’
Plgf-siRNA	5’-GGGAACAACUCAACAGAAATT-3’
Prkcd-siRNA	5′-GCUCAAGAGUGUCAAGAAATT-3′
negative siRNA	5’-UUCUCCGAACGUGUCACGUTT-3’

Supplementary Table 4 NetPhos 3.1-based prediction of candidate kinases in Ser^{304} of MTF-1

Phosphorylation site	Sequence	Score	Candidate kinase
304 S	STQYSLKSH	0.940	unsp
304 S	STQYSLKSH	0. 810	PKC
304 S	STQYSLKSH	0.577	CKI
304 S	STQYSLKSH	0.458	CaM-II
304 S	STQYSLKSH	0.450	GSK3
304 S	STQYSLKSH	0.357	DNAPK
304 S	STQYSLKSH	0.349	cdc2
304 S	STQYSLKSH	0.301	RSK
304 S	STQYSLKSH	0.290	PKG
304 S	STQYSLKSH	0.283	p38MAPK
304 S	STQYSLKSH	0.266	ATM
304 S	STQYSLKSH	0.264	CKII
304 S	STQYSLKSH	0.199	PKA
304 S	STQYSLKSH	0.187	cdk5
304 S	STQYSLKSH	0.081	PKB

Supplementary Table 5 PhosphoSitePlus-based prediction of candidate kinases in Ser^{304} of MTF-1

Kinase	Gene Name	Kinase Family	Log_2(Score)	Score Rank
PKCT	PRKCQ	AGC	2.6337	1
PHKG1	PHKG1	CAMK	2.4954	2
YSK4	MAP3K19	STE	2.3648	3
PKCG	PRKCG	AGC	2.3103	4
PKCB	PRKCB	AGC	2.1425	5
PKCH	PRKCH	AGC	2.094	6
PKCA	PRKCA	AGC	1.9933	7
MLK1	MAP3K9	TKL	1.7004	8
PHKG2	PHKG2	CAMK	1.6576	9
MLK3	MAP3K11	TKL	1.6255	10
PKCD	PRKCD	AGC	1.5606	11
NEK9	NEK9	Other	1.3733	12
IKKE	IKBKE	Other	1.3597	13
NEK2	NEK2	Other	1.2972	14
MTOR	MTOR	PIKK	1.2508	15
MLK4	MAP3K21	TKL	1.2317	16
DNAPK	PRKDC	PIKK	1.1303	17
MST4	STK26	STE	1.1219	18
IRE2	ERN2	Other	1.0912	19
MLK2	MAP3K10	TKL	1.064	20
MST3	STK24	STE	1.062	21
PKCE	PRKCE	AGC	1.0478	22
TBK1	TBK1	Other	1.0344	23
PKCI	PRKCI	AGC	1.0217	24
PKN2	PKN2	AGC	0.975	25
NEK11	NEK11	Other	0.9578	26
SLK	SLK	STE	0.9039	27
DLK	MAP3K12	TKL	0.8829	28
DRAK1	STK17A	CAMK	0.8694	29
RAF1	RAF1	TKL	0.8628	30
DSTYK	DSTYK	TKL	0.8579	31
PKN1	PKN1	AGC	0.8461	32
KHS1	MAP4K5	STE	0.8346	33
HPK1	MAP4K1	STE	0.8217	34
CAMK2D	CAMK2D	CAMK	0.804	35
NEK4	NEK4	Other	0.7723	36
BCKDK	BCKDK	PDHK	0.7688	37
MST2	STK3	STE	0.7642	38
TTBK2	TTBK2	CK1	0.7553	39

TAO3	TAOK3	STE	0.7462	40
MAPKAPK3	MAPKAPK3	CAMK	0.7274	41
ZAK	MAP3K20	TKL	0.7256	42
IRAK4	IRAK4	TKL	0.7236	43
BMPR2	BMPR2	TKL	0.719	44
TGFBR2	TGFBR2	TKL	0.7069	45
PKN3	PKN3	AGC	0.7069	45
NIK	MAP3K14	STE	0.7039	47
IKKA	CHUK	Other	0.6879	48
HRI	EIF2AK1	Other	0.6677	49
PKCZ	PRKCZ	AGC	0.6587	50

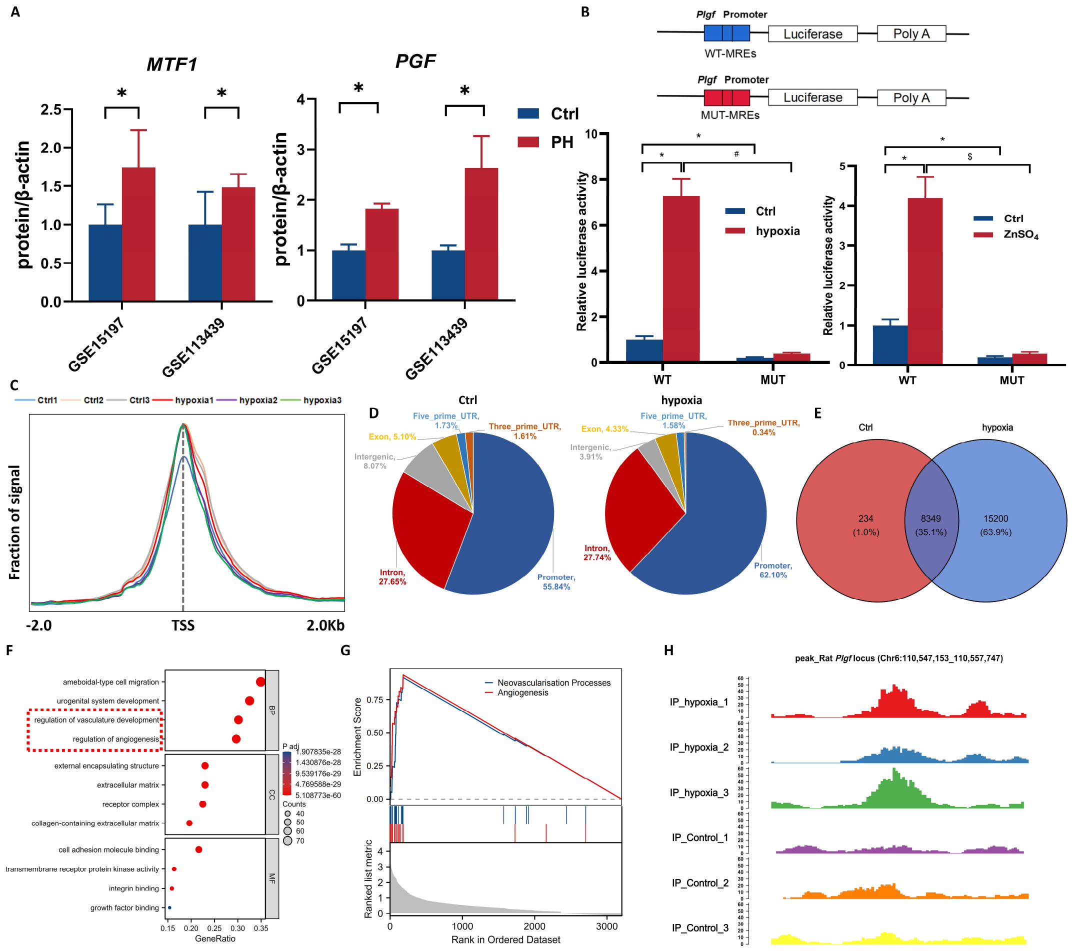

Supplementary Figure 1 Luciferase reporter assay and CUT&Tag analysis of MTF-1–dependent transcriptional activation of *Plgf*. **(A)** Quantification of *MTF1* and *PGF* expression in human lung tissues from GSE15197 and GSE113439. **(B)** Luciferase reporter assay for *Plgf* promoter constructs with wild-type or mutated MREs in hypoxia- or $ZnSO_4$-treated 293T cells. **(C)** Average signal intensity of CUT&Tag peaks centered on transcription start sites (TSS, ±2 kb). **(D)** Genomic distribution of CUT&Tag peaks. **(E)** Venn diagram showing the overlap of CUT&Tag peak-associated genes between control and hypoxia-treated samples. **(F-G)** GO enrichment analysis (F) and GSEA (G) of hypoxia-specific peaks. **(H)** Genome browser tracks showing CUT&Tag signal intensity across the *Plgf* locus (Chr6: 110,547,153–110,557,747). CUT&Tag: Cleavage Under Targets and Tagmentation; TSS: transcription start site; GO: Gene Ontology; GSEA: Gene Set Enrichment Analysis.

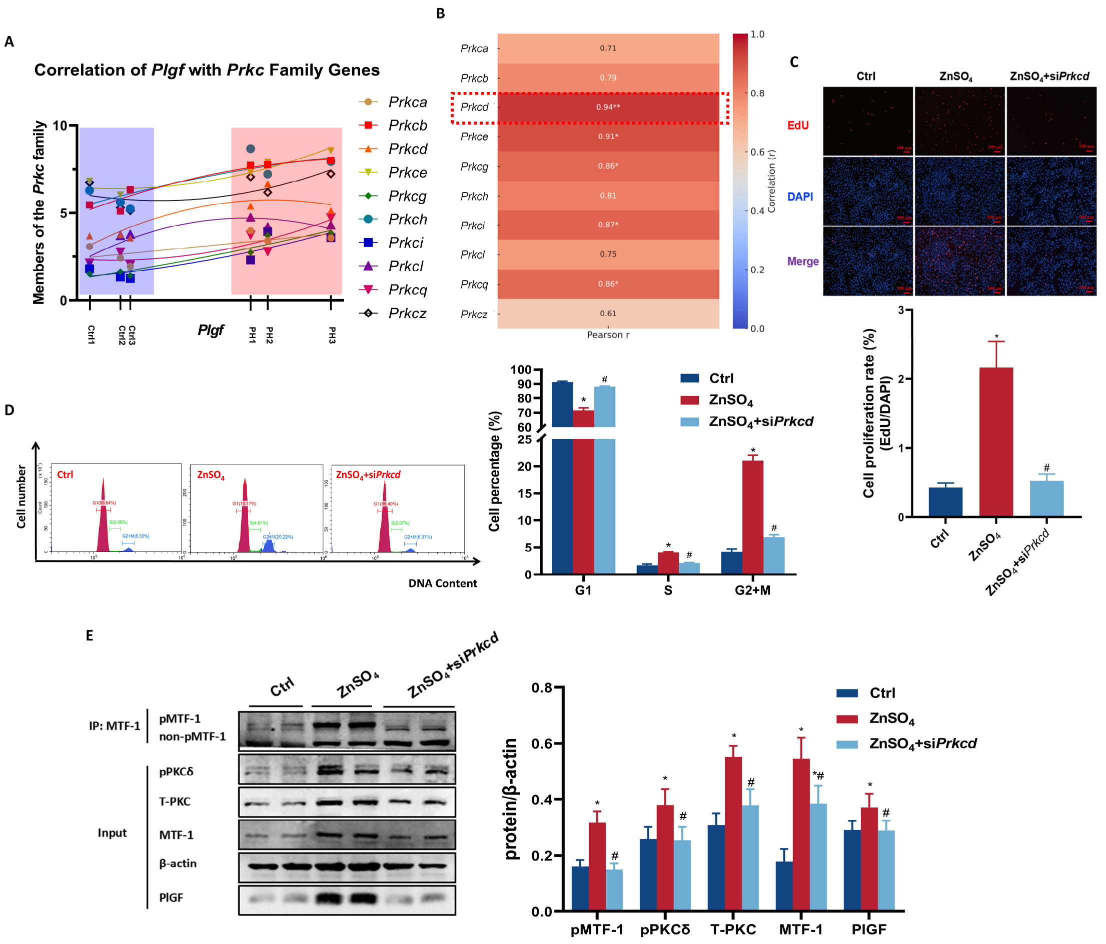

Supplementary Figure 2 Role of PKCδ kinase in zinc-induced MTF-1 phosphorylation. (A) Correlation between *Plgf* and members of the *Prkc* gene family in PH transcriptomic datasets from GSE186996. **(B)** Heatmap of correlation between *Plgf* and *Prkc* isoforms. **(C-E)** Effect of si*Prkcd* on proliferation of $ZnSO_4$-treated PASMCs detected by EdU assay (C), flow cytometry (D), and Western blot (E). EdU assay: scale bar=100 μm. Ctrl: control. $^*P$ <0.05 vs. Ctrl; $^\#P$ <0.05 vs. $ZnSO_4$. Data are represented as mean ± SD, n=5.
